# miR-105 Inhibits Prostate Tumour Growth by Suppressing CDK6 Levels

**DOI:** 10.1371/journal.pone.0070515

**Published:** 2013-08-07

**Authors:** D. Rice Honeywell, Miguel A. Cabrita, Huijun Zhao, Jim Dimitroulakos, Christina L. Addison

**Affiliations:** 1 Cancer Therapeutics Program, Ottawa Hospital Research Institute, Ottawa, Ontario, Canada; 2 Departments of Biochemistry Microbiology and Immunology, University of Ottawa, Ontario, Canada; 3 Department of Medicine, University of Ottawa, Ontario, Canada; Roswell Park Cancer Institute, United States of America

## Abstract

A significant role for micro (mi)RNA in the regulation of gene expression in tumours has been recently established. In order to further understand how miRNA expression may contribute to prostate tumour growth and progression, we evaluated expression of miRNA in two invasive prostate tumour lines, PC3 and DU145, and compared it to that in normal prostate epithelial cells. Although a number of miRNAs were differentially expressed, we focused our analysis on miR-105, a novel miRNA not previously linked to prostate cancer. miR-105 levels were significantly decreased in both tumour cell lines in comparison to normal prostate epithelial cells. To determine its potential role in prostate cancer pathogenesis, we overexpressed miR-105 in both PC3 and DU145 cells and determined its effect on various tumourigenic properties. miR-105 overexpression inhibited tumour cell proliferation, tumour growth in anchorage-independent three-dimensional conditions and tumour invasion in vitro, properties of highly aggressive tumour cells. Of potential clinical significance, miR-105 overexpression inhibited tumour growth *in vivo* in xenograft models using these cell lines. We further identified CDK6 as a putative target of miR-105 which is likely a main contributor to the inhibition of tumour cell growth observed in our assays. Our results suggest that miR-105 inhibits tumour cell proliferation and hence may represent a novel therapeutically relevant cellular target to inhibit tumour growth or a marker of aggressive tumours in prostate cancer patients.

## Introduction

Micro(mi) RNAs are small RNA inhibitors which have been shown to play an important role in regulating gene expression in a number of organisms. miRNAs were first discovered in *Caenorhabditis elegans* (C. *elegans*) [1], [2], and were subsequently shown to block gene expression by post-transcriptional binding and silencing of specific mRNA sequences. The transcription and processing of miRNA into its mature form has been recently described in extensive detail elsewhere [3]–[5]. Briefly, miRNA molecules are ∼19–22 nucleotides long and can be transcribed from either intergenic regions or within gene introns or exons [6]. Mature miRNA is then bound and unwound by the Argonaute protein and incorporated into the RISC, a protein complex which helps guide the mature miRNA strand to its target mRNA binding site [7] via a 5–7 nucleotide seed sequence within the mature miR strand [8]. After binding its target, the complex causes either inhibition of translation or degradation of the target mRNA molecule [9].

In recent years, miRNAs have been shown to play a role in many diseases, such as cancer [10], heart disease [11], and diabetes [12], [13]. Prostate cancer is no exception, and studies have shown that miRNA dysregulation occurs in the majority of prostate tumours [13]–[15]. Some of these dysregulated miRNAs have been shown to modulate the prostate cancer phenotype by affecting crucial growth regulatory targets such as K-Ras [16], androgen receptor [17] and cyclin D1 [18]. Other miRNAs, for example, miR-21, have been dubbed ‘oncomirs’ as a result of their overexpression leading to the promotion of tumour phenotypes in numerous cancer types [19]–[21]. There are also miRNAs that function as tumour suppressors, and inhibit the tumourigenic phenotype in cancer cells. For example, miR-143 is downregulated in many cancers, as it has been shown to regulate, and allow expression of genes that promote proliferation and migration, such as K-Ras and cyclinD1 [16]. Moreover, modulating cellular miRNA expression using depletion strategies [22] or overexpression of miRNAs [23], can regulate tumour phenotype both *in vitro* and *in vivo.* Thus increasing our understanding of miRNA dysregulation in prostate cancer will facilitate our understanding of prostate cancer progression and could also identify important novel therapeutic targets and predictive miRNA signatures for advanced and/or aggressive disease.

Given their important role in modulating the tumourigenic phenotype, we set out to identify miRNAs that play a significant role in mediating growth and invasion of aggressive prostate tumour cells. To this end, we performed a miRNA microarray experiment to compare the miRNA expression profiles of normal prostate epithelial cells (PrEC) with those of two well characterized metastatic prostate tumour cell lines (PC3 and DU145). Although a number of significant differences were uncovered, we focused our study on determining the role of differentially expressed miRNAs not previously reported to regulate prostate tumour progression. Hence, we examined the role of miR-105 in this study, which demonstrated significantly decreased expression in both metastatic prostate tumour cell lines when compared to PrEC.

miR-105 belongs to a group of miRs whose levels are regulated in cancer cells by preventing export of their Drosha/DGCR8 processed pre-miR forms from the nucleus into the cytoplasm [24]. Overexpression of miR-105 was also shown to be associated with changes in proliferation markers in primary ovarian granulosa cells [25]. Given these observations together with our array data, we evaluated the role of miR-105 as a potential novel regulator of prostate cancer cell proliferation, anchorage-independent growth and invasion *in vitro* and tumour growth of xenografted prostate cancer cells *in vivo*. Overall our data suggests that miR-105 inhibits growth of prostate tumours both *in vitro* and *in vivo*.

## Materials and Methods

### Cells and media

PC3 and DU145 human prostate carcinoma cells were obtained from ATCC (Manassas, VA). These cells were grown in Dulbecco's Modified Eagle Medium (DMEM, Mediatech Inc, Manassas, VA) supplemented with 10% Fetal Bovine Serum (FBS). PrEC (Normal Prostate Epithelial Cells) were obtained from Lonza (Walkersville, MD), and were grown in PrEGM (Lonza, Walkersville, MD) for expansion. PrEC were only used between passages 4–10. All cells were grown at 37°C in 5% CO_2_ in a humidified chamber. For three-dimensional (3D) anchorage independent growth assays, 2× alpha MEM was obtained from Gibco, Grand Island, NY).

### Cell Transfection

Cells were seeded at an ∼40–50% density, and following adherence overnight were transfected with miRIDIAN hsa-miR-105 specific mimic or the cel-miR-67 control mimic miRNAs (all from Dharmacon, Lafayette, CO) using Oligofectamine™ (Invitrogen, Carlsbad, CA) in Opti-MEM® (Invitrogen, Carlsbad, CA) as described by the manufacturer. Cel-miR-67 is based on a sequence for a *C. elegans* miRNA that shows no sequence similarities to any human, mouse or rat genomic sequences as analyzed by BLAST searches, nor any effect on human miRNA function (www.dharmacon.com). It has been used as an effective control for miRNA overexpression or inhibition by a number of previous studies [26]–[28]. For reduction of miR-105 levels in cells, miRIDIAN hairpin inhibitors to miR-105 or control hairpin inhibitors (to cel-miR-67) were used (both from Dharmacon, Lafayette CO) and were transfected into cells using similar approaches. Transfection efficiencies in all cases were monitored by fluorescence of the tagged control miRNA and by quantitative reverse transcription polymerase chain reaction (qRT-PCR) for specific miRNAs as described below.

### Microarrays

Genechip® Human Gene 1.0 ST and Genechip® microRNA microarrays (Affymetrix, Santa Clara, CA) were used for microarray analysis of samples. RNA was isolated from the cell lines using the miRNeasy kit (Qiagen, Valencia, CA) according to the manufacturer's instructions. mRNA samples for the Genechip® Human Gene 1.0 ST were prepared using the Whole-transcript Sense Target Labeling Assay (Affymetrix, Santa Clara, CA). miRNA samples for the Genechip® microRNA microarrays were prepared with the Flashtag™ Biotin HSR kit (Genisphere, Hatfield, PA). Prepared samples were then sent to Stemcore Laboratories (Ottawa, ON) for microarray processing. Microarray data were analyzed using the FlexArray software package [29] (URL http://genomequebec.mcgill.ca/FlexArray). Annotation files of chip compositions were obtained from the Affymetrix website (www.affymetrix.com) and were used to generate target lists for Human Gene microarray data.

### Quantitative real time-polymerase chain reaction (qRT-PCR)

For qRT-PCR, total RNA containing small RNAs was prepared from either human cancer cells or tumour xenograft tissue using the miRNeasy kit (Qiagen, Valencia, CA). Total RNA concentration was assessed using a Nanodrop Spectrophotometer (Thermo Scientific, Wilmington, DE). For miRNAs, RNA samples (diluted to 5 ng/ul in nuclease-free water) were reverse transcribed using the Taqman® microRNA Reverse Transcription Kit (Applied Biosystems, Carlsbad, CA) as per manufacturer's instructions, using specific miRNA primers (TaqMan primer sets for hsa-miR-103: Cat. #4427975, RNU24: Cat. #4427975, or hsa-miR-105: Cat. #4427975 were used). cDNAs were then amplified by qPCR using miRNA-specific TaqMan primers in a 7500 Fast Real-Time PCR system (Applied Biosystems, Carlsbad, CA). Results were normalized to the levels of amplified hsa-miR-103 or RNU24 as endogenous controls and fold differences calculated using the 7500 FAST software (version 2.0, Applied Biosciences).

For analysis of mRNA, total RNA samples were reverse transcribed using random hexamers and MMLV Reverse Transcriptase (Invitrogen, Carlsbad, CA). For qRT-PCR, specific amplicons for β-actin and CDK6 were generated using RT^2^ Fast SYBR® Green/Rox™ qPCR Master Mix (SABiosciences, Frederick, MD) in conjunction with the following primer pairs: β-actin, Forward: 5′-GGGCATGGGTCAGAAGGAT-3′, Reverse: 5′-GTGGCCATCTCTTGCTCGA-3′; Cdk6, Forward: 5′-TGATCAACTAGGAAAAATCTTGGAC-3′ Reverse: 5′-GGCAACATCTCTAGGCCAGT-3′ using an ABI 7500 FAST Real Time PCR System. All primers were designed using the online Roche primer design tool (https://www.roche-applied-science.com/sis/rtpcr/upl/index.jsp?id=UP030000). The results were analyzed using the 7500 FAST software (version 2.0) and the levels of CDK6 were normalized to β-actin.

### MTT assays

Cells were transfected with miRNA mimics or hairpin inhibitors as described above, and seeded into 96-well plates at 2500 cells per well. For tumour cell lines, MTT solution (3-(4,5-dimethylthiazol-2-yl)-2,5-diphenyltetrazolium bromide) was added at 24 hour intervals to wells for 3–4 hours, and subsequently 0.01 N HCl in 10% SDS lysis solution was added. The plate was then incubated overnight and absorbance was read at 570 nm in a Multiskan Ascent plate reader (Thermo Scientific, Rockford, IL). For PrEC, due to their slow population doubling time, Alamar Blue reagent (Invitrogen, Mississauga ON) was instead used according to the manufacturer's directions and absorbance measured similarly.

### Apoptosis assays

Cells were transfected as described above. Twenty-four hours post-transfection, cells were placed in serum-free media. After 48 hours (72 hours post-transfection), cells were collected for either analysis by flow cytometry or immunoblot. For flow cytometry, non-adherent cells were pooled with trypsinized adherent cells from each dish, washed with cold PBS and then fixed and permeabilized in 100% ethanol by incubation at −20°C for a minimum of 24 hours. Cells were again washed in PBS, followed by staining with propidium iodide (PI) solution (48 µg/ml propidium iodide, 40 µg/ml RNase A in PBS) for 30 minutes at 4°C. Samples were then analyzed using a Coulter EPICS XL flow cytometer (Beckman-Coulter, Mississauga, ON) on the FL2 channel. The percentage of apoptotic cells with less than 2 N DNA content was determined using FCS Express flow cytometry analysis software (De Novo Software, Los Angeles, CA). The proportion of cells in G1 and G2/M was determined using ModFit LT (Verity Software House, Topsham, ME).

For western blot analysis, total protein lysates from harvested cells (adherent and non-adherent) were generated following lysis in Frack's lysis buffer as described in detail below. Western blots were performed as described below, to visualize the cleavage of PARP as an indication of the degree of apoptosis occurring in treated cells.

### Anchorage-independent three-dimensional (3D) growth assays

Cells were transfected as described. Twenty-four hours after transfection, cells were resuspended at various dilutions in a 1∶1 mixture of 1% agarose with 2× alpha-MEM +20% FBS and were overlayed on a base layer composed of a 1∶1 mixture of 1.5% agarose with 2× alpha-MEM +20% FBS in 24 well plates. The agarose mixtures were allowed to solidify briefly at room temperature until they became viscous, at which time they were placed at 37°C for 14 days. Two weeks later, representative photos were taken of each well using a Nikon digital camera on an Olympus CK2 inverted phase-contrast microscope at 100× magnification, which were then assessed for colony number and size.

### Scratch wound assays

Twenty-four hours post-transfection cells were seeded into 6-well plates at a density that would ensure cell confluency the next day. Cell monolayers were then scratched using a sterile wounding tool, and 4 images/well were acquired with a digital camera (Nikon, Mississauga, ON) at 0 and 48 hour time points at 40× magnification using an Eclipse TE2000-U microscope (Nikon). Wound diameters in images were measured and percentage wound closure was calculated as follows: [(wound diameter at 48 hours – wound diameter at 0 hours)/wound diameter at 0 hours] ×100.

### Invasion assays

Cells were transfected as described above. The next day, cells were harvested and placed in serum free DMEM in the top chamber of a Matrigel-coated transwell (BD Biocoat growth-factor reduced Matrigel Invasion Chamber, PET membranes, Mississauga, ON), and allowed to migrate towards serum containing DMEM in the bottom well for 24 hours. Afterwards, the Matrigel and all non-invaded cells were removed and invasive cells on the underside of the transwell filter were visualized by crystal violet (0.5% crystal violet solution in 25% methanol). Invasive cells were then quantified following microscopic quantification at 100× magnification, across the entire membrane. Results are expressed as the mean total invaded cells/well in duplicate wells, for three pooled independent experiments.

### In vivo tumour xenograft models

Five-week old CD1 nude mice were purchased from Charles River Laboratories (Wilmington, MA) and housed in the Animal Care and Veterinary Services facility at the University of Ottawa. All protocols performed using mouse models were approved by the University of Ottawa Animal Care Committee and conformed to the guidelines set by the *Animals for Research Act* and the Canadian Council on Animal Care's *Guide to the Care and Use of Experimental Animals*, Vol. 1, 2nd ed., 1993) and meet standards of practice in the disciplines of laboratory animal science and laboratory animal veterinary medicine.

For xenograft models, transfected PC3 or DU145 cells were harvested 24 hours post-transfection, and 1×10^6^ cells in 150 uL of sterile PBS were injected subcutaneously into both hind flanks of the mice. Tumour measurements were taken weekly following caliper measurements of visible subcutaneous tumours. Volume was calculated using the formula [Volume  =  (width)^2^× (length/2)]. At necropsy, dissected tumours were measured in three dimensions to calculate tumour volume, wet tumour weight was obtained, and tumour tissue samples were obtained and immediately fixed in 10% formalin solution (Sigma, St. Louis, MO) for histological analysis. In most cases, RNA from a portion of each tumour was extracted, and levels of miR-105 were quantified as described above. Levels of miR-105 expression were normalized to endogenous levels of RNU24, and then subsequently normalized to the average miR-105 level of control mimic treated tumour samples in each case.

### Antibodies and Western Immunoblotting

Total protein from cells was generated following lysis in Frack's buffer (10mM Tris-HCl, pH 7.4, 150 mM NaCl, 5 mM EDTA, 1% Triton X-100, ddH_2_O) and sonication using a Sonicator 3000 (Misonix, Newtown, CT). The homogenized samples were then centrifuged in a microfuge for 30 minutes at 4°C and cleared supernatants were transferred to fresh tubes and stored at −20°C. For western blot analysis, equal amounts of total protein were separated on 10% SDS-PAGE gels. Proteins were then transferred to Hybond™ -C Extra nitrocellulose membranes (GE Healthcare, Piscataway, NJ), and membranes blocked using 5% skim milk powder in Tris buffered saline with Tween (TBST; 10 mM Tris pH 8.0, 150 mM NaCl, 0.05% Tween 20; herein referred to as 5% milk) or 5% bovine serum albumin (BSA) in TBST (herein referred to as 5% BSA) for 1 hour. Specific proteins were subsequently detected following incubation in primary antibody diluted in 5% milk or 5% BSA overnight at 4°C. Monoclonal mouse anti-β-actin antibody from Sigma-Aldrich (St. Louis, MO) was used in western blots at a 1∶5,000–1∶10,000 dilution. Monoclonal rabbit anti-Cdk6 (Novus Biologicals, Littleton, CO) was used at a 1∶500 dilution, and polyclonal rabbit anti-PARP (Cell Signaling, Danvers, MA) was used at a 1∶1,000 dilution in western blot analysis. Horse-radish peroxidase (HRP) conjugated secondary antibodies to mouse or rabbit IgG were obtained from Sigma and used at 1∶5,000 or 1∶10,000 dilutions for visualization of bound primary antibodies in western blots. Following incubation in primary antibody, blots were incubated in diluted HRP-conjugated secondary antibody at room temperature for 1 hour. Proteins were then visualized using the Immobilon Western chemiluminescent HRP substrate (Millipore, Billerica, MA) and exposing the blots to film (HyBlot CL autoradiography sheets, Denville Scientific, Metuchen, NJ). For re-probing of blots, membranes were stripped using Restore PLUS Western Blot stripping buffer (Thermo Scientific, Rockford, IL), re-blocked and then re-probed with additional antibodies.

### Graphical and Statistical Analysis

All data was plotted using GraphPad Prism software (version 3.02, GraphPad Software, San Diego, CA). Statistical differences for single comparison tests were determined using two-tailed unpaired t-test with a 95% confidence interval. For multiple comparisons a non-parametric one-way ANOVA test with a 95% confidence interval was used. Tests were performed using the statistical programs within the GraphPad Prism software (version 3.02, GraphPad Software, San Diego, CA). Results with a p-value of less than 0.05 were deemed significant.

## Results

### miR-105 expression is decreased in prostate cancer cell lines compared to normal prostate epithelium

**Figure 1:**
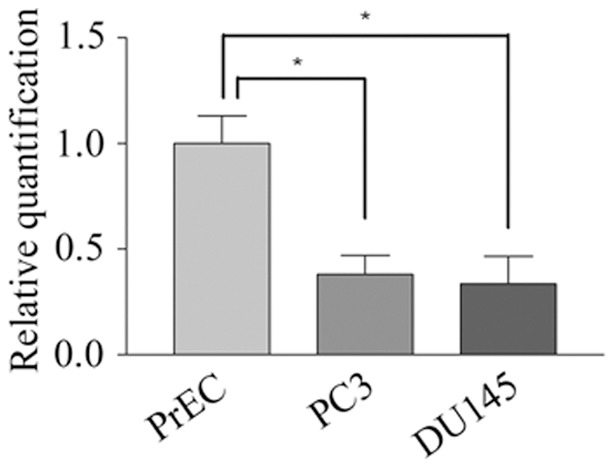
miR-105 levels are reduced in prostate tumour cells compared to normal prostate epithelial cells. Levels of miR-105 were quantified in PrEC, PC3 and DU145 using TaqMan qRT-PCR. Relative levels of miR-105 were normalized to endogenous levels of RNU24 as a control. Graphs represent the pooled mean and standard error of 8 replicates for each cell line from two independent experiments. The relative levels of miR-105 were significantly decreased in PC3 and DU145 cells compared to normal PrEC cells (* represents p<0.05).

We compared miRNA expression of metastatic prostate tumour cells (PC3 and DU145) with that of normal prostate epithelial cells (PrEC) using Affymetrix chip profiling as described in the materials and methods. Although we initially only performed microarrays with a sample size of 1 for each cell type, a significant number of miRNAs were found to be differentially expressed in both tumour cell lines as compared to PrEC, including many miRNAs already shown to be associated with prostate cancer such as miR-205 [30], miR-200 [31] and miR-146a [32]. We subsequently validated differentially expressed miRNA targets, however as the role of many of these miRNAs in cancer had been previously described, we were more interested in determining the function of miRNAs whose oncogenic role had not previously been defined. Thus, we focused our efforts on miR-105, and determined its levels were approximately 62% and 67% lower in the tumour cell lines PC3 and DU145 respectively as compared to in PrEC following analysis by qRT-PCR (Fig. 1). In this assay, levels of miR-105 were normalized to endogenous levels of RNU24 or miR-103, as these two miRNAs have been previously shown to be stably expressed in both tumour and normal cells and thus represented appropriate normalization controls [33], [34]. Given that miR-105 is reduced in both metastatic prostate tumour cell lines compared to normal prostate epithelium, we hypothesized that miR-105 expression may inhibit prostate tumour cell growth or invasion.

### Characterization of miR-105 overexpression in prostate tumour cell lines

**Figure 2:**
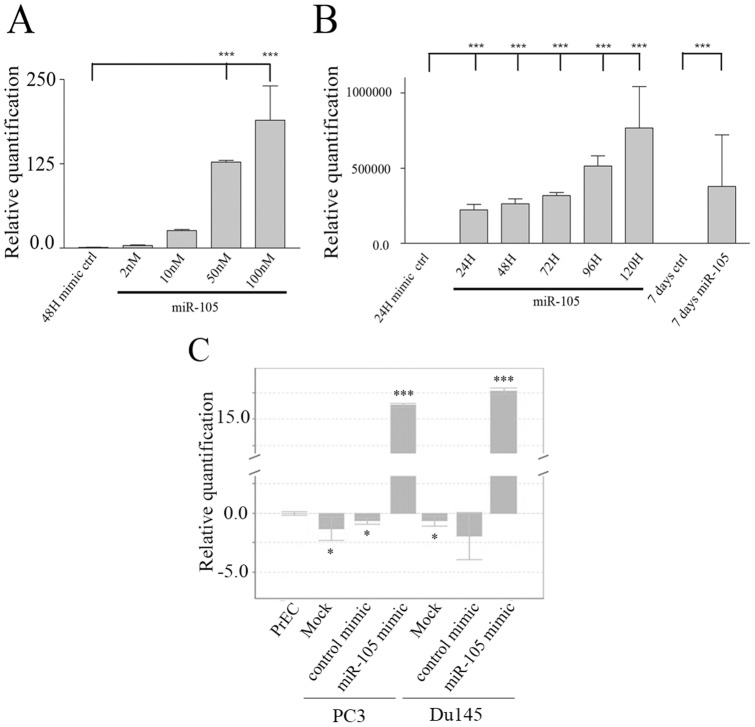
Evaluation of efficacy of MIRIDIAN mimics for modulation of miR-105 levels. (A) PC3 cells were treated with increasing concentrations (2, 10, 50, 100 nM) of miR-105 specific or 100 nM control (cel-miR-67) mimics (Dharmacon). After 48 hours samples were evaluated for relative levels of miR-105 using TaqMan qRT-PCR. miR-105 levels specifically increased in a dose-dependent fashion in miR-105 compared to control mimic transfected cells. The graph represents the mean and standard error of 8 replicates from one experiment. Similar trends were observed in other independent experiments. (B) PC3 cells were treated with 50 nM of miR-105 or control cel-miR-67 mimics, and relative levels of expression of miR-105 were assessed using TaqMan qRT-PCR. miR-105 expression increased over time, and peaked at 120 hours post-transfection before declining, while miR-105 levels remained similar in control mimic-transfected cells over time. In all cases levels were normalized to endogenous levels of RNU24 as an internal control. The graph represents the mean and standard error of 3 replicates from one experiment. Similar results were observed in other independent experiments. *** represents p<0.001 as compared to control mimic-transfected cells for panels A and B. (C) Levels of miR-105 were determined in untransfected PrEC, PC3 or DU145 cells and compared to levels in PC3 or DU145 cells transfected with 50 nM miR-105 or cel-miR-67 mimics. In all cases levels were normalized to endogenous levels of RNU24 as an internal control, and to endogenous levels in PrEC. Bars represent the mean and standard error of 3 replicates from one experiment. Similar results were observed in other independent experiments. * represents p<0.05 and *** represents p<0.001.

To study the effects of modulation of miR-105 expression on various aspects of tumourigenesis, we used MIRIDIAN miRNA mimics (Dharmacon). Hsa-miR-105 and cel-miR-67 (which has no known homology to human sequences) were used as the specific and control mimic miRNAs, respectively. The most effective mimic concentration resulting in significantly increased miR-105 levels was determined following transfection of different concentrations (2 nM, 10 nM, 50 nM, and 100 nM) of mimics and quantification of the level of miR-105 expression in transfected cells using qRT-PCR. We found that 50 nM of miR-105 mimic was sufficient to significantly overexpress miR-105 in PC3 (Fig. 2A) and DU145 (data not shown) cells as compared to control mimic transfected cells. We also tested the duration of miR-105 overexpression in PC3 cells following transfection of 50 nM of miR-105 or control mimics, and measured miR-105 levels over 7 days post-transfection (Fig. 2B). miR-105 expression peaked at 5 days post-transfection of mimic, and began to decline at 7 days but still persisted at relatively high levels. Control mimic-treated cells showed no change in expression of miR-105 levels over the same time course. We also compared miR-105 levels in mimic transfected PC3 and DU145 cells to the endogenous levels of miR-105 in PrEC (Fig. 2C). Tumour cells transfected with 50 nM miR-105 mimic expressed approximately 15-fold higher levels of miR-105 than that found endogenously in normal PrEC.

### miR-105 overexpression suppresses tumour cell growth *in vitro*

**Figure 3:**
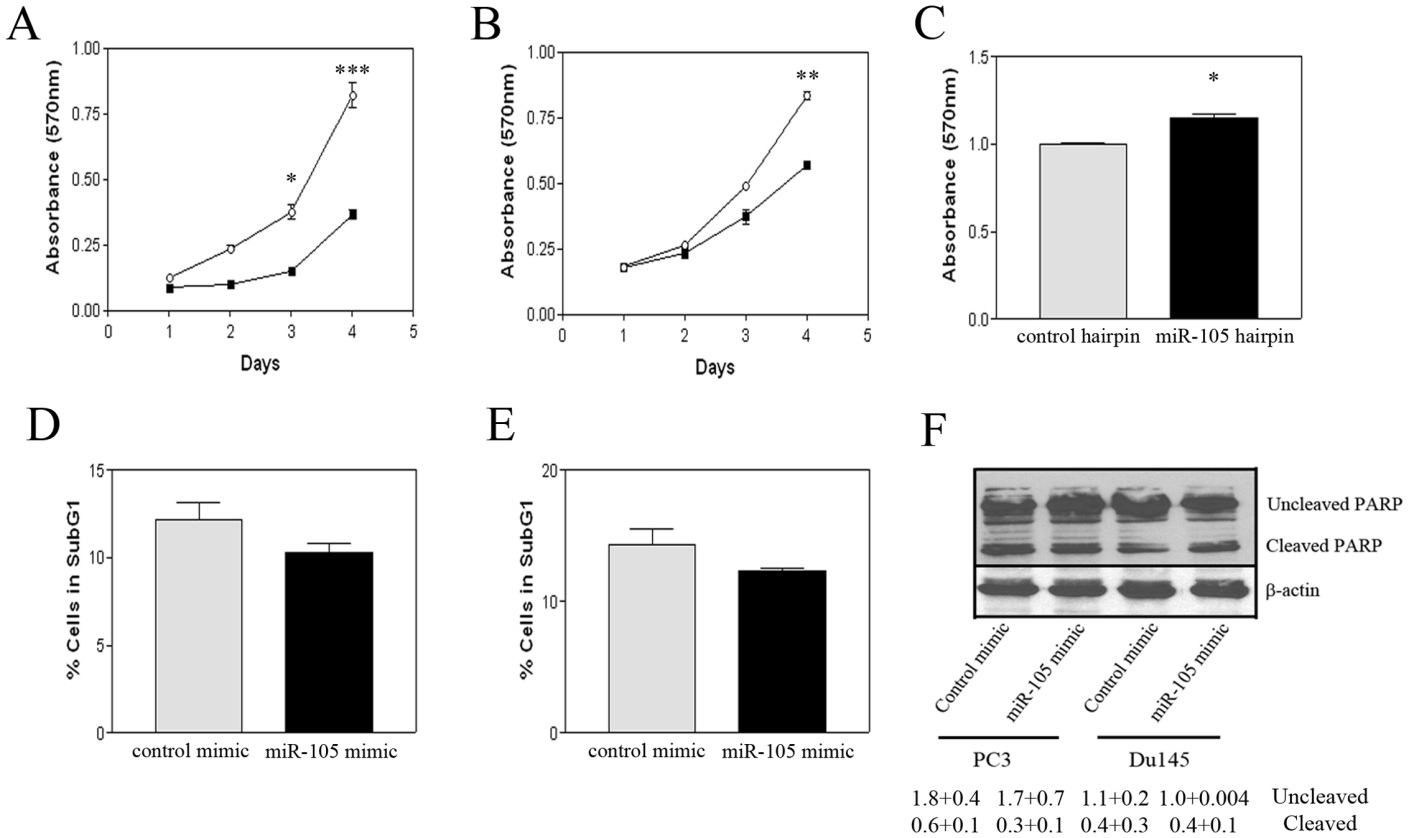
Overexpression of miR-105 inhibits tumour cell growth but does not induce apoptosis. (A–B) Tumour cells were transfected with either 50 nM miR-105 (closed squares) or control (open circles) mimics and subsequently seeded into 96-well plates in replicates of 8. Cell viability was assessed over time using the MTT assay as described in materials and methods. Graphs represent the pooled mean and standard error of 8 replicates from three independent experiments. Both miR-105 overexpressing PC3 (A) and DU145 (B) cells showed a significant decrease in cell number/viability over time (* represents p<0.05, ** represents p<0.01 and *** represents p<0.001 respectively) as compared to control mimic transfected cells. (C) PrEC were transfected with 50 nM of miR-105 or control hairpin inhibitors, and 24 hours later, equal numbers of transfected cells were seeded in 96 well microtiter plates. Cell growth was assessed 48 hours later as described in materials and methods. A modest increase in the mean number of viable cells (plotted with associated standard error) was observed. Graphs represent the pooled mean and standard error of 8 replicates in each of two independent experiments. (D–F) To analyze apoptosis, cells were transfected with 50 nM of miR-105 or control mimic (50 nM) as described in materials and methods and then serum-starved for an additional 48 hours. Apoptotic cells were determined as the percentage cells in sub-G1 as assessed by flow cytometry of propidium iodide stained cells as described. Bars represent the pooled mean and standard error of triplicate dishes from two independent experiments. There was no significant difference in the number of apoptotic cells between the miR-105 and control mimic-treated populations for either PC3 (D) or DU145 (E) cells. (F) Serum starved cells as described above, were also analyzed for PARP cleavage by specific western blot analysis. There were no significant differences in the levels of cleaved PARP noted between the miR-105 and control mimic-transfected cell populations. β-actin was used as a loading control for equivalent amounts of total protein. Mean intensity of bands in western blots (n = 2) and associated standard error for uncleaved and cleaved PARP relative to β-actin levels are indicated below image.

In order to evaluate the effects of miR-105 overexpression on tumourigenic properties, we transfected prostate tumour cell lines with miR-105-specific or control mimics at 50 nM. In the various assays described below, cells were used to initiate experiments at 24 hours post-transfection, a concentration and time-point whereby readily detectable levels of miR-105 overexpression were observed (Fig. 2B). We initially examined the effect of miR-105 overexpression on tumour cell growth and viability in PC3 (Fig. 3A) and DU145 (Fig. 3B) cells using the MTT cell viability assay. Cell viability was assessed over time, and at 96 hours post-seeding (120 hours post-transfection) we found that elevated miR-105 levels reduced the relative viable cell number by 55% and 31% for PC3 and DU145 respectively, when compared to control mimic-overexpressing cells. Thus miR-105 overexpression inhibits prostate tumour cell growth.

To support our findings, we also assessed whether depletion of miR-105 in normal PrEC altered their growth or viability. Following transfection with 50 nM miR-105 hairpin inhibitor, we observed that the growth of PrEC was modestly enhanced following inhibition of endogenous miR-105 (Fig. 3C). These findings thus support the role of miR-105 as an inhibitor of cell growth.

### miR-105 overexpression does not affect prostate tumour cell apoptosis

As cell number/viability was assessed by MTT assay, the reduction observed following miR-105 overexpression could be due to either increased levels of apoptosis or decreased cell proliferation. To determine whether miR-105 overexpression induced apoptosis in PC3 cells, we used two standard methods. Levels of apoptosis induced following serum withdrawal were determined following detection of the subG1 proportion of propidium iodide stained cells (Fig. 3D and 3E for PC3 and DU145 cells, respectively). Furthermore, apoptosis of cells was also assessed following evaluation of PARP cleavage by Western blot (Fig 3F). No significant differences were observed in the levels of cell apoptosis when miR-105 was overexpressed as measured by either method under either normal growth conditions (data not shown) or under conditions of serum starvation (Fig. 3D–F). These data suggest that the reduced viable cell numbers observed in the MTT assays were more likely the result of inhibition of tumour cell growth and proliferation over time. We also assessed the percentage of cells in various stages of the cell cycle using the estimation program ModFit as described in materials and methods. We observed a slight decrease in the number of serum starved (for 48 hours) miR-105 expressing PC3 cells in G1 phase (46+3% vs 37+3% for control vs. miR-105 mimic) with a concomitant increase in the number of cells in G2 (52+2% vs. 62+2% for control vs miR-105 mimic) and no significant changes in S-phase (2+1% vs. 1+1%). This suggests that although no increases in apoptosis may result from miR-105 overexpression, PC3 cell growth may become arrested in G2. In DU145 cells, miR-105 expression resulted in slight decreases in G1 (64+1% vs. 61+2%), and G2 (36+0.3% vs. 30+2%), however, slight increases in the number of cells in S-phase (0.2+0.2% vs. 9+4%) were apparent. As none of these differences were found to be statistically significant, taken together our results suggest that there is no significant effect of miR-105 of the regulation of apoptosis in prostate tumour cells.

### miR-105 over-expression suppresses anchorage-independent growth *in vitro*

**Figure 4:**
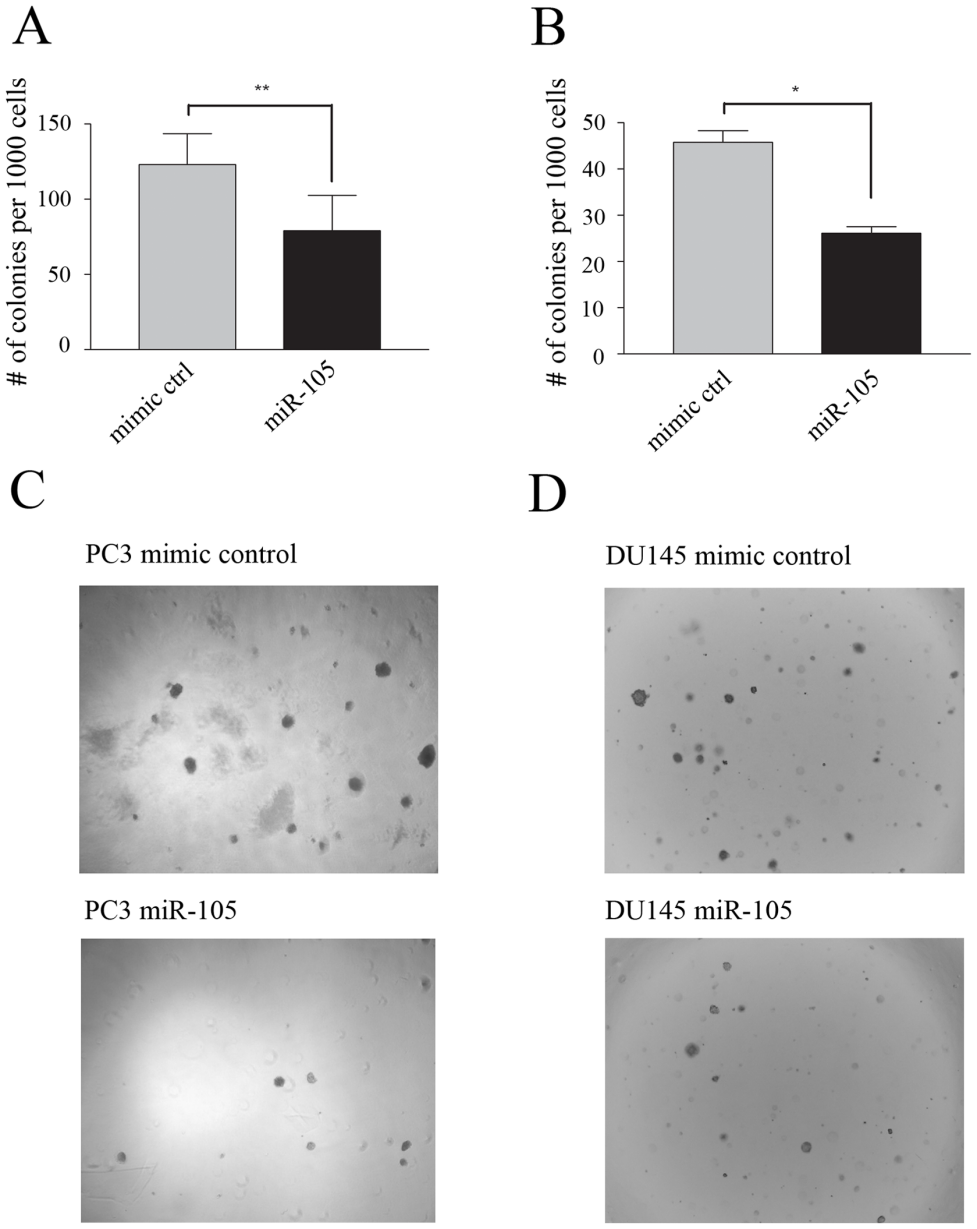
Overexpression of miR-105 mimic inhibits anchorage-independent growth of prostate tumour cells. (A–D) PC3 (A) and DU145 (B) cells were transfected with either 50 nM miR-105 or control mimic and 24 hours post-transfection were seeded in soft-agarose. The number of colonies was then counted in triplicate wells generated from the 1000 cell/well dilution. Bars represent the pooled mean and standard error for triplicate wells from three independent experiments. There was a significant decrease in colony formation in cells treated with miR-105 as compared to control mimic-transfected cells for both PC3 (** represents p<0.01) and DU145 (* represents p<0.05) cells. (C–D) Representative photos showing reduced colony formation in soft-agarose for miR-105 versus control mimic-transfected PC3 (C) and DU145 (D) cells.

We also tested the effect of miR-105 overexpression on prostate tumour cell growth in anchorage-independent 3D conditions using soft agarose assays. Following a two week incubation period, the number of colonies was counted in each well. The ability of PC3 (Fig. 4A&C) or DU145 (Fig. 4B&D) cells overexpressing miR-105 to form colonies in 3D was reduced by 36% and 43% respectively, when compared to cells transfected with control mimics. Taken together, our results suggest that increased levels of miR-105 suppress tumour cell growth in both 2D and 3D growth conditions.

### Overexpression of miR-105 inhibits tumour cell migration and invasion

**Figure 5:**
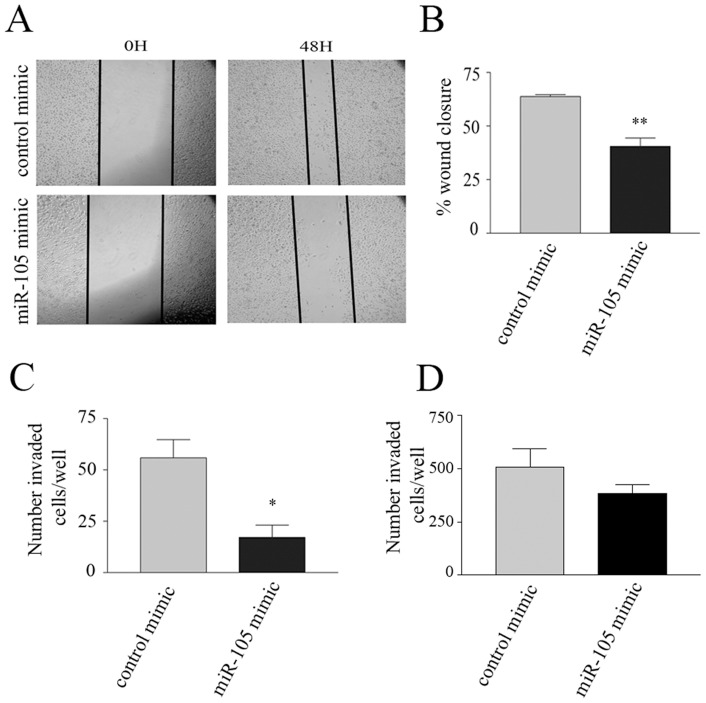
Overexpression of miR-105 mimic inhibits tumour cell migration and invasion. (A) Representative photos of scratch wound assays at 0 and 48 hours post wounding for miR-105 compared to control mimic-transfected PC3 cells showing impaired wound closure in miR-105 mimic-overexpressing cells. Photos were taken at 40× magnification. (B) PC3 migration was measured as percentage wound closure in assays in (A) as described in materials and methods. Average wound closure measurements at three different points along wound edge were quantified in each of two independent wells. Bars represent pooled mean and standard error of duplicate dishes in each of two independent experiments. Wound closure after 48 hours was significantly less (** represents p<0.01) in the miR-105 mimic-expressing compared to control mimic-transfected cells. (C) PC3 or (D) DU145 cell invasion was measured over 24 hours using Matrigel-coated transwells as described. The total number of invasive cells on the underside of each filter were counted and expressed in the graph as the pooled mean and standard error from duplicate wells in three independent experiments. There were significantly less (* represents p<0.05) miR-105 mimic-overexpressing invaded PC3 cells compared to control mimic-transfected cells, and a similar trend was observed for DU145 cells.

We next evaluated whether miR-105 overexpression affected tumour cell migration and invasion. To test this, miR-105 transfected cells were used in a standard scratch wound assay. We found that the migration of miR-105 mimic-treated PC3 cells over a 48 hour time period was significantly inhibited compared to control mimic-transfected cells (Fig. 5A and 5B). In contrast, we did not observe any significant inhibition in the migration of miR-105 transfected DU145 cells in the scratch wound assay (data not shown). As wound closure rates can be influenced by cell proliferation, and we had previously noted that miR-105 overexpression significantly inhibited PC3 cell growth and moderately inhibited DU145 cell growth, we investigated the ability of miR-105 to inhibit tumour cell invasion using Matrigel-coated transwell invasion chambers. Since this is a short term assay, cell proliferation should not play a significant role, and any observed effects will mostly be a result of direct impact on cell migration and invasion through Matrigel. In this assay, we found that the number of invasive PC3 cells transfected with miR-105 mimics was significantly reduced as compared to control mimic-transfected cells (Fig. 5C). We also observed a trend in miR-105 transfected DU145 cells to be less invasive than mimic control transfected cells (Fig. 5D). Thus, overexpression of miR-105 also appears to inhibit tumour cell migration and invasion.

### Overexpression of miR-105 inhibits subcutaneous growth of prostate tumour cells *in vivo*

**Figure 6:**
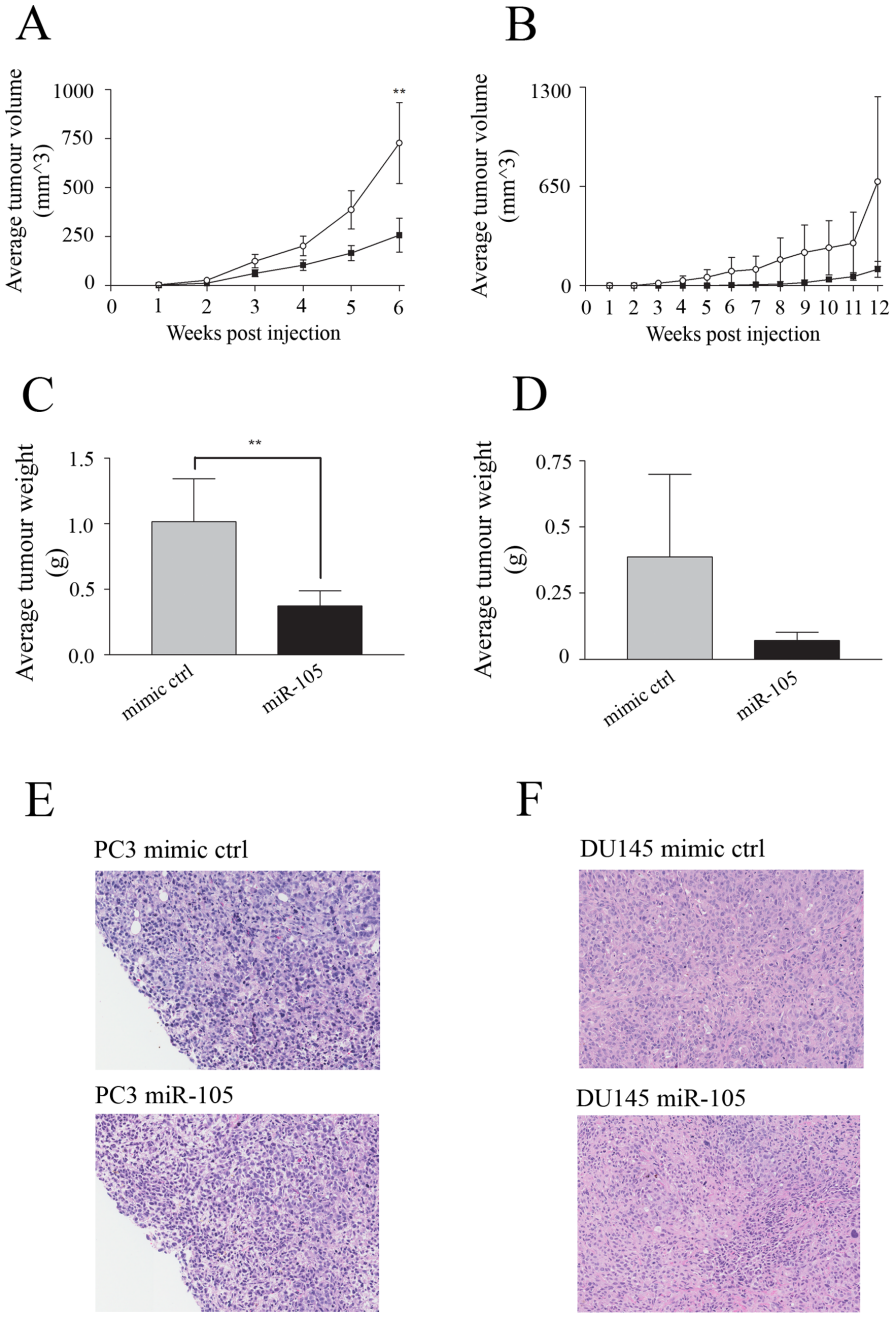
Overexpression of miR-105 inhibits *in vivo* subcutaneous tumour growth. (A–G) PC3 and DU145 cells were transfected with either 50 nM miR-105 or control mimics and injected subcutaneously in the hind flanks of 5-week old CD1 nude mice as described in materials and methods. For each condition, data is representative of 16 bilateral tumours from 8 mice. (A–B) Tumour size was measured by calipers over time for PC3 (A) and DU145 (B) derived tumours until humane endpoints were reached. Tumours derived from miR-105 overexpressing PC3 cells were significantly smaller than tumours derived from control mimic-transfected PC3 cells while tumours derived from miR-105 mimic-transfected DU145 cells trended to be smaller than their respective controls. (C–D) Tumour wet weight at experimental endpoint was also measured for PC3 (C) and DU145 (D) derived tumours. The mean weight in grams and associated standard error is plotted, and shows that at endpoint miR-105 mimic-transfected PC3 were significantly smaller compared with control mimic-transfected cells. DU145 tumours showed similar trends but did not quite reach statistical significance. (E–F) At endpoint tumour sections were histologically analyzed. No significant differences in tumour morphology were observed between miR-105 versus control mimic-transfected cell derived tumours for either PC3 (E) or DU145 (F) tumours. In all cases ** represents p<0.01.

We next examined the effects of miR-105 overexpression on *in vivo* tumour xenograft growth in immunocompromised mice. PC3 or DU145 cells transfected with either miR-105 specific or control mimics were harvested 24 hours later, and 1×10^6^ cells were injected subcutaneously into each flank of immunocompromised nude mice. Tumour growth was measured weekly, and tumour volumes were assessed for PC3 (Fig. 6A) and DU145 (Fig. 6B) tumour growth over time. We found that the growth of PC3 tumours was significantly inhibited following overexpression of miR-105 mimics when compared to control mimic-transfected PC3 cells (Fig. 6A). At the termination of the experiment, wet tumour weight was also determined, and it was confirmed that miR-105 overexpressing tumours weighed on average 73% less than the control tumours (Fig. 6C). Although not statistically significant, we also observed reduced DU145 tumour growth in miR-105 transfected as compared to controls over a longer time course (Fig. 6B). At endpoint, the weight of tumours derived from miR-105 transfected DU145 cells was approximately 82% less than those derived from control mimic transfected cells (Fig. 6D). It should be noted however, that DU145 derived tumours had a much longer latency and were overall much smaller than PC3 cell derived tumours which likely contributed to only an inhibitory trend being observed in the growth inhibition noted between miR-105 and control mimic-expressing xenografted tumour cells. In addition, histological staining of tumour sections from both PC3 (Fig. 6E) and DU145 (Fig. 6F) derived tumours showed no major cellular or morphological differences between control and miR-105 mimic-treated cells. At experimental endpoint, total RNA was extracted from tumour samples and the levels of miR-105 expression were assessed using qRT-PCR. Not surprisingly, similar levels of miR-105 expression were observed in all tumours analyzed at endpoint (relative fold differences of 1.0±0.6 for control and 2.2±1.5 for miR-105 mimic-transfected PC3 derived tumours). Similar results were observed for DU145 (data not shown). These results support the contention that miR-105 overexpression inhibits prostate tumour cell growth resulting in delayed tumour progression.

### miR-105 targets Cdk6 in prostate tumour cells

**Figure 7:**
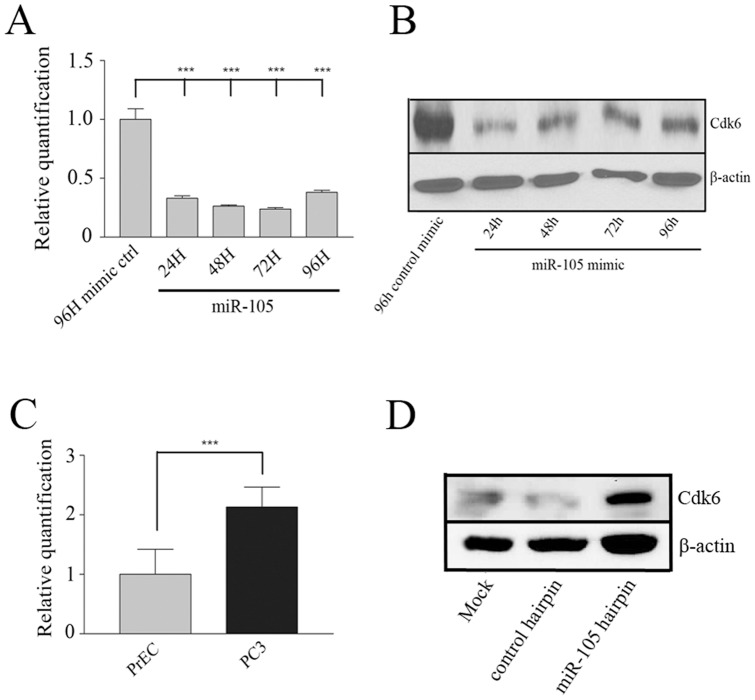
miR-105 results in decreased Cdk6 expression at both the mRNA and protein levels. (A–B) PC3 cells were transfected with 50 nM miR-105 or control mimics as described in materials and methods and relative levels of Cdk6 expression were assessed over time. Both the mRNA and protein samples were isolated from paired dishes of transfected cells for each condition. (A) Relative levels of CDK6 mRNA were determined following qRT-PCR analysis and normalization to endogenous levels of β-actin as a control. The graph represents the mean and standard error of triplicate dishes from one experiment. Similar trends were observed in other independent experiments. *** represents p<0.001. (B) Cdk6 protein levels were assessed by western blot analysis. Blots were reprobed for levels of β-actin as a loading control for total protein. We observed a significant decrease in both Cdk6 mRNA and protein expression in miR-105 overexpressing as compared to control mimic-transfected PC3 cells. Blots were repeated a minimum of three independent times. (C) CDK6 mRNA levels were also compared between normal PrEC cells and cancerous PC3 cells using qRT-PCR and normalization to endogenous β-actin levels as a control. Consistent with expressing reduced endogenous miR-105 levels, PC3 cells had higher CDK6 expression when compared to PrEC. The graph represents the mean and standard error of triplicate samples from one experiment. Similar trends were observed in other independent experiments. *** represents p<0.001. (D) PrEC were either mock transfected or transfected with hsa-miR-105 or cel-miR-67 hairpin inhibitors, and Cdk6 levels assessed by western blot analysis. Blots were reprobed for β-actin as a loading control for total protein. Cdk6 levels were significantly elevated in PrEC transfected with miR-105 as compared to controls. Blots were repeated two independent times with similar results.

To determine the mechanism through which miR-105 overexpression was inhibiting tumour growth and invasion, we attempted to identify relevant mRNA targets of miR-105. To do this, we compared the gene expression profiles of PC3 cells transfected with either the miR-105 or control mimic using Affymetrix GeneChip Human Gene 1.0 ST microarrays (n = 1). As we presumed that the main mechanism of miR-105 modulation of the observed cell phenotypes was via down-regulation of specific gene products, we were particularly interested in targets that were down-regulated in miR-105 overexpressing cells as compared to control mimic-transfected cells. Our top down-regulated target was CDK6, an important cell-cycle regulatory protein, and a likely candidate to explain the reduced 2D and 3D growth we previously observed. We thus validated the microarray findings using qRT-PCR for CDK6 message. We found that CDK6 expression was significantly reduced in PC3 cells treated with 50 nM of miR-105 compared to control mimic-transfected cells over 96 hours (Fig. 7A). To confirm these findings, we also examined the protein levels of Cdk6 by immunoblot analysis. We found that the level of Cdk6 protein was also reduced in PC3 cells transfected with miR-105 over 96 hours as compared to control mimic-transfected cells (Fig. 7B), thus confirming our results and suggesting that Cdk6 is a target of miR-105. As we had originally determined that miR-105 was higher in normal prostate epithelial cells as compared to tumour cells, we examined the relative expression of CDK6 between normal PrEC cells and PC3 tumour cells. Consistent with our results, we found that the message levels of CDK6 were elevated in the PC3 cell line as compared to PrEC (Fig. 7C), further demonstrating the inverse correlation between miR-105 and CDK6 expression. This was also observed at the protein level following transfection of PrEC with miR-105 hairpin inhibitors. As seen in Fig. 7D, PrEC transfected with miR-105 hairpin inhibitors had elevated Cdk6 expression compared to control cells, again supporting the notion that miR-105 is able to target CDK6 message. Taken together our results suggest that miR-105 inhibits prostate tumour cell growth in part via its ability to downregulate the expression of the important cell cycle regulator CDK6.

## Discussion

In this study we found that miR-105 overexpression inhibits the growth of both PC3 and DU145 prostate cancer cells *in vitro* and *in vivo*. We further found that the inhibition of prostate tumour growth may be attributable to the ability of miR-105 to reduce CDK6 levels, which is also supported by our data using hairpin inhibitors whereby reduced miR-105 levels in PrEC resulted in increased Cdk6 expression and enhanced growth in those cells. Cdk6 is an important cell-cycle regulator protein that acts with Cdk4 to phosphorylate and inactivate the Retinoblastoma (Rb) protein to allow cell cycle progression [35]. It regulates the transition from G1 to S phase and its dysregulation is important in many cancer types, including prostate [36] and breast cancer [37]. In cancer it has been shown that many miRs, such as miR-107 [38], miR-129 [39], and miR-449a/b [40], target and down-regulate CDK6 to reduce cell proliferation and invasion. It is possible that miR-105 affects CDK6 directly, as it is a predicted target of miR-105 by the online miR target prediction program Targetscan (http://www.targetscan.org/cgi-bin/targetscan/vert_61/view_gene.cgi?taxid=9606&rs=NM_001145306&members=miR-105/105ab&showcnc=1&shownc=1&showncf=1), which predicts 4 seed regions in the 3′ UTR of CDK6, namely 287–293, 538–544, 2625–2632 and 7613–7619. However, it also remains possible that miR-105 downregulation of CDK6 could be via indirect mechanism in our studies, which requires further investigation. The downregulation of CDK6 following miR-105 mimic overexpression in tumour cells would explain the effects on cell proliferation and invasion we see in our experiments. Also, the inverse relationship between the CDK6 and miR-105 expression levels we observed between PrEC and the prostate tumour cell lines (Fig. 7C), together with the increase in Cdk6 expression in PrEC transfected with miR-105 hairpin inhibitors (Fig. 7D) lends further support to our hypothesis. We also observed that miR-105 overexpression impaired prostate tumour cell migration and invasion, although the mechanism by which this effect is mediated remains to be elucidated. However, it has been previously shown that Cdk6 can modulate integrin-mediated cell spreading and migration [41], hence miR-105 targeting of CDK6 could also in part explain the migration and invasion defects we observed.

Generally, we observed similar findings in both PC3 and DU145 cells, with the exception of cell migration as assessed by scratch wound assays and the *in vivo* studies. In the in vivo experiments, although we observed a trend for tumours derived from miR-105 mimic-expressing DU145 to be smaller than control mimic-expressing tumours, the differences were not statistically significant. This is not surprising as we found that growth of DU145 in our xenograft models was significantly slower than that of PC3 tumours (experimental endpoints of 12 weeks compared to 7 weeks respectively). Given that we used transient overexpression of miR mimics (which appear to decrease 7–14 days post-transfection), it is likely that the overexpression of miR-105 in DU145 tumours *in vivo* using this method was not sufficiently durable to demonstrate significant growth differences over the extended time period of tumour growth. Despite this, we are encouraged by the fact that even transient overexpression of miR-105 in this manner was sufficient to inhibit tumour growth, which further supports the important role of miR-105 in prostate tumour growth. It is also possible that PC3 cells are more dependent on Cdk6 activity to suppress the function of Rb protein in order to proliferate as they retain wild-type Rb expression. In contrast, DU145 cells express a mutant Rb protein which is already inactive, thus the role of Cdk6 in mediating their growth is likely less crucial, and could hence account for the more modest influences of miR-105 modulation in DU145 cells we observed in our studies. This possibility is supported by the fact that many studies have recently demonstrated that tumours which are most sensitive to Cdk4/6 inhibitors are proficient for Rb [42], [43], suggesting that these kinases drive tumour proliferation in the context of wild-type Rb activity.

Our study of miR-105 focused on its expression levels and effects in prostate cancer. However, other cancer cell lines have been shown to have decreased mature miR-105 expression, including HS766T, MiaPaca2 and Panc1 (pancreatic carcinomas), HCT-116 (colorectal carcinoma), HeLa (cervical carcinoma), and HL-60 (promyelocytic leukemia) [24]. In addition, we found that miR-105 overexpression in A549 lung carcinoma cells caused a significant decrease in cell proliferation and anchorage-independent growth (data not shown). Thus it is possible that miR-105 may play a more general role in inhibiting tumour growth in multiple cancer types, in particular those which express wild-type Rb protein. One question raised by these results is the mechanism of the repression of miR-105 levels in cancer cells. It has been suggested that the mature form of miR-105 is undetectable in certain cancer cell lines compared with abundant precursor miR-105 molecules present in the nucleus of these same cells [24]. This could be attributed to the fact that some cancers have lower levels of functional exportin-5, an important miR biogenesis protein [44]. Exportin-5 mediates pre-miRNA nuclear transport into the cytoplasm, and some cancers possess loss-of-function mutations in this gene, which inhibits mature miRNA production. It has been shown that PC3 cells have lower levels of exportin-5 than normal prostate cells (RWPE) [45]. Thus it is possible that the lower levels of exportin-5 in metastatic prostate tumour cells lead to decreases in the mature miR-105 levels that we would have quantified using the Taqman microRNA assay system.

Our findings suggest that miR-105 is a new addition to a growing list of at least 200 miRs that are dysregulated in prostate cancer [14], and will lead to better understanding of how the disease progresses from initiation to metastasis. To date there have been multiple studies that examine the unique miR profiles associated with different cancer types, including prostate cancer. One of the largest microarray analysis to date by Shaefer *et al*., reported ten miRs that were significantly downregulated and five miRs that were upregulated in malignant prostate tissue compared to matched normal prostate tissue [46]. However, miR-105 was not one of the downregulated targets reported in their study. One reason for this could be due to the fact that they compared paired normal and tumour samples from patient primary tumours, and these cohorts were predominantly newly diagnosed lower risk, non-metastatic patients. As the two cell lines we used in our study were both derived from secondary metastatic prostate tumours, specifically, from a lumbar vertebrae for PC3 cells [47], and from the central nervous system for DU145 cells [48], it remains possible that miR-105 downregulation is associated with acquisition of a more metastatic phenotype. The microarray results of Shaefer *et al.* are also confounded by the fact that the investigators did not microdissect prostate tumour tissue away from intervening normal stromal cells. As prostate tumours are very heterogeneous, the relative levels of expression of the various miRNAs could be masked by the contribution of the stromal levels of miR-105 that may remain elevated.

We have recently queried the NCBI GEO datasets using the GEO2R analysis function for additional miRNA array data in prostate cancer. In support of our findings, a study comparing miRNA expression in the same cell types we examined (DU145 and PC3) found that miR-105 levels were 70% lower in the prostate tumour lines compared to the normal prostate epithelial cell line RPWE (GSE17317) [49]. Moreover, the relevance to human prostate cancer has been more recently demonstrated by Wach et al., whereby prostate tumour epithelium was microdissected away from other stromal cells prior to analysis. In this dataset, levels of miR-105 were found to be decreased by 6-fold in the prostate tumour epithelium as compared to microdissected adjacent normal prostate tissue (GSE23022) [50]. Additionally, miR-105 levels were 2.4-fold lower in the urine of prostate cancer patients as compared to those with benign prostatic hyperplasia, again supporting the contention that its levels are decreased as prostate cancer progresses (GSE39314). Also in support of our findings, a recent meta-analysis of miRNA expression datasets from hepatocellular carcinomas suggested that downregulation of miR-105 was a common feature [51], thus investigations into its role in human prostate cancer are warranted.

Overall our findings suggest that miR-105 is a novel factor that regulates prostate tumour growth and that is likely to play a role in other cancer types. It joins a growing list of miRs that have a direct impact on cancer proliferation, anchorage-independent growth and invasion, and hopefully in the future will have applications for direct targeted miRNA therapies and in predictive biomarker applications.

## References

[1] Lee RC, Feinbaum RL, Ambros V (1993) The C. elegans heterochronic gene lin-4 encodes small RNAs with antisense complementarity to lin-14. Cell 75: 843–854. 8252621 10.1016/0092-8674(93)90529-y

[2] Wightman B, Ha I, Ruvkun G (1993) Posttranscriptional regulation of the heterochronic gene lin-14 by lin-4 mediates temporal pattern formation in C. elegans. Cell 75: 855–862. 8252622 10.1016/0092-8674(93)90530-4

[3] Winter J, Jung S, Keller S, Gregory RI, Diederichs S (2009) Many roads to maturity: microRNA biogenesis pathways and their regulation. Nat Cell Biol 11: 228–234. 19255566 10.1038/ncb0309-228

[4] Lee Y, Ahn C, Han J, Choi H, Kim J, et al (2003) The nuclear RNase III Drosha initiates microRNA processing. Nature 425: 415–419. 14508493 10.1038/nature01957

[5] Lund E, Guttinger S, Calado A, Dahlberg JE, Kutay U (2004) Nuclear export of microRNA precursors. Science 303: 95–98. 14631048 10.1126/science.1090599

[6] Lee Y, Kim M, Han J, Yeom KH, Lee S, et al (2004) MicroRNA genes are transcribed by RNA polymerase II. EMBO J 23: 4051–4060. 15372072 10.1038/sj.emboj.7600385 PMC524334

[7] Gregory RI, Chendrimada TP, Shiekhattar R (2006) MicroRNA biogenesis: isolation and characterization of the microprocessor complex. Methods Mol Biol 342: 33–47. 16957365 10.1385/1-59745-123-1:33

[8] Lee I, Ajay SS, Yook JI, Kim HS, Hong SH, et al (2009) New class of microRNA targets containing simultaneous 5′-UTR and 3′-UTR interaction sites. Genome Res 19: 1175–1183. 19336450 10.1101/gr.089367.108 PMC2704433

[9] He L, Hannon GJ (2004) MicroRNAs: small RNAs with a big role in gene regulation. Nat Rev Genet 5: 522–531. 15211354 10.1038/nrg1379

[10] Calin GA, Croce CM (2006) MicroRNA-cancer connection: the beginning of a new tale. Cancer Res 66: 7390–7394. 16885332 10.1158/0008-5472.CAN-06-0800

[11] van Rooij E, Olson EN (2007) MicroRNAs: powerful new regulators of heart disease and provocative therapeutic targets. J Clin Invest 117: 2369–2376. 17786230 10.1172/JCI33099 PMC1952642

[12] Kolfschoten IG, Roggli E, Nesca V, Regazzi R (2009) Role and therapeutic potential of microRNAs in diabetes. Diabetes Obes Metab 11 Suppl 4118–129. 10.1111/j.1463-1326.2009.01118.x 19817794

[13] Sevli S, Uzumcu A, Solak M, Ittmann M, Ozen M (2010) The function of microRNAs, small but potent molecules, in human prostate cancer. Prostate Cancer Prostatic Dis 13: 208–217. 20585343 10.1038/pcan.2010.21

[14] Gordanpour A, Nam RK, Sugar L, Seth A (2012) MicroRNAs in prostate cancer: from biomarkers to molecularly-based therapeutics. Prostate Cancer Prostatic Dis. 10.1038/pcan.2012.3 22333688

[15] Hassan O, Ahmad A, Sethi S, Sarkar FH (2012) Recent updates on the role of microRNAs in prostate cancer. J Hematol Oncol 5: 9. 22417299 10.1186/1756-8722-5-9 PMC3313897

[16] Xu B, Niu X, Zhang X, Tao J, Wu D, et al (2011) miR-143 decreases prostate cancer cells proliferation and migration and enhances their sensitivity to docetaxel through suppression of KRAS. Mol Cell Biochem 350: 207–213. 21197560 10.1007/s11010-010-0700-6

[17] Sikand K, Slaibi JE, Singh R, Slane SD, Shukla GC (2011) miR 488* inhibits androgen receptor expression in prostate carcinoma cells. Int J Cancer 129: 810–819. 21710544 10.1002/ijc.25753 PMC3839820

[18] Aqeilan RI, Calin GA, Croce CM (2010) miR-15a and miR-16-1 in cancer: discovery, function and future perspectives. Cell Death Differ 17: 215–220. 19498445 10.1038/cdd.2009.69

[19] Ribas J, Ni X, Haffner M, Wentzel EA, Salmasi AH, et al (2009) miR-21: an androgen receptor-regulated microRNA that promotes hormone-dependent and hormone-independent prostate cancer growth. Cancer Res 69: 7165–7169. 19738047 10.1158/0008-5472.CAN-09-1448 PMC2861586

[20] Corcoran C, Friel AM, Duffy MJ, Crown J, O'Driscoll L (2011) Intracellular and extracellular microRNAs in breast cancer. Clin Chem 57: 18–32. 21059829 10.1373/clinchem.2010.150730

[21] Gao W, Xu J, Liu L, Shen H, Zeng H, et al (2012) A systematic-analysis of predicted miR-21 targets identifies a signature for lung cancer. Biomed Pharmacother 66: 21–28. 22244963 10.1016/j.biopha.2011.09.004

[22] Dong CG, Wu WK, Feng SY, Wang XJ, Shao JF, et al.. (2012) Co-inhibition of microRNA-10b and microRNA-21 exerts synergistic inhibition on the proliferation and invasion of human glioma cells. Int J Oncol. 10.3892/ijo.2012.1542 22766763

[23] Bader AG (2012) miR-34 – a microRNA replacement therapy is headed to the clinic. Front Genet 3: 120. 22783274 10.3389/fgene.2012.00120 PMC3387671

[24] Lee EJ, Baek M, Gusev Y, Brackett DJ, Nuovo GJ, et al (2008) Systematic evaluation of microRNA processing patterns in tissues, cell lines, and tumors. RNA 14: 35–42. 18025253 10.1261/rna.804508 PMC2151027

[25] Sirotkin AV, Laukova M, Ovcharenko D, Brenaut P, Mlyncek M (2010) Identification of microRNAs controlling human ovarian cell proliferation and apoptosis. J Cell Physiol 223: 49–56. 20039279 10.1002/jcp.21999

[26] Mazar J, Khaitan D, DeBlasio D, Zhong C, Govindarajan SS, et al (2011) Epigenetic regulation of microRNA genes and the role of miR-34b in cell invasion and motility in human melanoma. PLoS One 6: e24922. 21949788 10.1371/journal.pone.0024922 PMC3176288

[27] Tie J, Pan Y, Zhao L, Wu K, Liu J, et al (2010) MiR-218 inhibits invasion and metastasis of gastric cancer by targeting the Robo1 receptor. PLoS Genet 6: e1000879. 20300657 10.1371/journal.pgen.1000879 PMC2837402

[28] Kole AJ, Swahari V, Hammond SM, Deshmukh M (2011) miR-29b is activated during neuronal maturation and targets BH3-only genes to restrict apoptosis. Genes Dev 25: 125–130. 21245165 10.1101/gad.1975411 PMC3022258

[29] Blazejczyk M, Miron M, Nadon R (2007) FlexArray: A statistical data analysis software for gene expression microarrays. Montreal, Canada: Genome Quebec.

[30] Gandellini P, Folini M, Longoni N, Pennati M, Binda M, et al (2009) miR-205 Exerts tumor-suppressive functions in human prostate through down-regulation of protein kinase Cepsilon. Cancer Res 69: 2287–2295. 19244118 10.1158/0008-5472.CAN-08-2894

[31] Kong D, Li Y, Wang Z, Banerjee S, Ahmad A, et al (2009) miR-200 regulates PDGF-D-mediated epithelial-mesenchymal transition, adhesion, and invasion of prostate cancer cells. Stem Cells 27: 1712–1721. 19544444 10.1002/stem.101 PMC3400149

[32] Lin SL, Chiang A, Chang D, Ying SY (2008) Loss of mir-146a function in hormone-refractory prostate cancer. RNA 14: 417–424. 18174313 10.1261/rna.874808 PMC2248249

[33] Carlsson J, Helenius G, Karlsson M, Lubovac Z, Andren O, et al (2010) Validation of suitable endogenous control genes for expression studies of miRNA in prostate cancer tissues. Cancer Genet Cytogenet 202: 71–75. 20875868 10.1016/j.cancergencyto.2010.06.009

[34] Peltier HJ, Latham GJ (2008) Normalization of microRNA expression levels in quantitative RT-PCR assays: identification of suitable reference RNA targets in normal and cancerous human solid tissues. RNA 14: 844–852. 18375788 10.1261/rna.939908 PMC2327352

[35] Ortega S, Malumbres M, Barbacid M (2002) Cyclin D-dependent kinases, INK4 inhibitors and cancer. Biochim Biophys Acta 1602: 73–87. 11960696 10.1016/s0304-419x(02)00037-9

[36] Lim JT, Mansukhani M, Weinstein IB (2005) Cyclin-dependent kinase 6 associates with the androgen receptor and enhances its transcriptional activity in prostate cancer cells. Proc Natl Acad Sci U S A 102: 5156–5161. 15790678 10.1073/pnas.0501203102 PMC556011

[37] Lucas JJ, Domenico J, Gelfand EW (2004) Cyclin-dependent kinase 6 inhibits proliferation of human mammary epithelial cells. Mol Cancer Res 2: 105–114. 14985467

[38] Lee KH, Lotterman C, Karikari C, Omura N, Feldmann G, et al (2009) Epigenetic silencing of MicroRNA miR-107 regulates cyclin-dependent kinase 6 expression in pancreatic cancer. Pancreatology 9: 293–301. 19407485 10.1159/000186051 PMC2835374

[39] Wu J, Qian J, Li C, Kwok L, Cheng F, et al (2010) miR-129 regulates cell proliferation by downregulating Cdk6 expression. Cell Cycle 9: 1809–1818. 20404570 10.4161/cc.9.9.11535

[40] Yang X, Feng M, Jiang X, Wu Z, Li Z, et al (2009) miR-449a and miR-449b are direct transcriptional targets of E2F1 and negatively regulate pRb-E2F1 activity through a feedback loop by targeting CDK6 and CDC25A. Genes Dev 23: 2388–2393. 19833767 10.1101/gad.1819009 PMC2764491

[41] Fahraeus R, Lane DP (1999) The p16(INK4a) tumour suppressor protein inhibits alphavbeta3 integrin-mediated cell spreading on vitronectin by blocking PKC-dependent localization of alphavbeta3 to focal contacts. EMBO J 18: 2106–2118. 10205165 10.1093/emboj/18.8.2106 PMC1171295

[42] Dean JL, McClendon AK, Hickey TE, Butler LM, Tilley WD, et al (2012) Therapeutic response to CDK4/6 inhibition in breast cancer defined by ex vivo analyses of human tumors. Cell Cycle 11: 2756–2761. 22767154 10.4161/cc.21195 PMC3409015

[43] Konecny GE, Winterhoff B, Kolarova T, Qi J, Manivong K, et al (2011) Expression of p16 and retinoblastoma determines response to CDK4/6 inhibition in ovarian cancer. Clin Cancer Res 17: 1591–1602. 21278246 10.1158/1078-0432.CCR-10-2307 PMC4598646

[44] Melo SA, Moutinho C, Ropero S, Calin GA, Rossi S, et al (2010) A genetic defect in exportin-5 traps precursor microRNAs in the nucleus of cancer cells. Cancer Cell 18: 303–315. 20951941 10.1016/j.ccr.2010.09.007

[45] Yi R, Doehle BP, Qin Y, Macara IG, Cullen BR (2005) Overexpression of exportin 5 enhances RNA interference mediated by short hairpin RNAs and microRNAs. RNA 11: 220–226. 15613540 10.1261/rna.7233305 PMC1370710

[46] Schaefer A, Jung M, Mollenkopf HJ, Wagner I, Stephan C, et al (2010) Diagnostic and prognostic implications of microRNA profiling in prostate carcinoma. Int J Cancer 126: 1166–1176. 19676045 10.1002/ijc.24827

[47] Kaighn ME, Narayan KS, Ohnuki Y, Lechner JF, Jones LW (1979) Establishment and characterization of a human prostatic carcinoma cell line (PC-3). Invest Urol 17: 16–23. 447482

[48] Mickey DD, Stone KR, Wunderli H, Mickey GH, Paulson DF (1980) Characterization of a human prostate adenocarcinoma cell line (DU 145) as a monolayer culture and as a solid tumor in athymic mice. Prog Clin Biol Res 37: 67–84. 7384095

[49] Boll K, Reiche K, Kasack K, Morbt N, Kretzschmar AK, et al (2013) MiR-130a, miR-203 and miR-205 jointly repress key oncogenic pathways and are downregulated in prostate carcinoma. Oncogene 32: 277–285. 22391564 10.1038/onc.2012.55

[50] Wach S, Nolte E, Szczyrba J, Stohr R, Hartmann A, et al (2012) MicroRNA profiles of prostate carcinoma detected by multiplatform microRNA screening. Int J Cancer 130: 611–621. 21400514 10.1002/ijc.26064

[51] Wang W, Peng B, Wang D, Ma X, Jiang D, et al (2011) Human tumor microRNA signatures derived from large-scale oligonucleotide microarray datasets. Int J Cancer 129: 1624–1634. 21128228 10.1002/ijc.25818

